# Experimental Robot Model Adjustments Based on Force–Torque Sensor Information

**DOI:** 10.3390/s18030836

**Published:** 2018-03-11

**Authors:** Santiago Martinez, Juan Miguel Garcia-Haro, Juan G. Victores, Alberto Jardon, Carlos Balaguer

**Affiliations:** System Engineering and Automation Department, University Carlos III, Av de la Universidad, 30, Madrid 28911, Spain; jgarciah@ing.uc3m.es (J.M.G.-H.); jcgvicto@ing.uc3m.es (J.G.V.); ajardon@ing.uc3m.es (A.J.); carlos.balaguer@uc3m.es (C.B.)

**Keywords:** force–torque sensors, balance control, humanoid robot, simplified model

## Abstract

The computational complexity of humanoid robot balance control is reduced through the application of simplified kinematics and dynamics models. However, these simplifications lead to the introduction of errors that add to other inherent electro-mechanic inaccuracies and affect the robotic system. Linear control systems deal with these inaccuracies if they operate around a specific working point but are less precise if they do not. This work presents a model improvement based on the Linear Inverted Pendulum Model (LIPM) to be applied in a non-linear control system. The aim is to minimize the control error and reduce robot oscillations for multiple working points. The new model, named the Dynamic LIPM (DLIPM), is used to plan the robot behavior with respect to changes in the balance status denoted by the zero moment point (ZMP). Thanks to the use of information from force–torque sensors, an experimental procedure has been applied to characterize the inaccuracies and introduce them into the new model. The experiments consist of balance perturbations similar to those of push-recovery trials, in which step-shaped ZMP variations are produced. The results show that the responses of the robot with respect to balance perturbations are more precise and the mechanical oscillations are reduced without comprising robot dynamics.

## 1. Introduction

In robotics, the most versatile but complex machines are humanoid robots. Their complex mechanical structure, high number of degrees of freedom (DOFs), and control requirements result in the need for simplifications that enable the deployment of multiple tasks. Human-like or humanoid robots are designed to work in scenarios in the same way that humans do, but at present they have very serious limitations when performing certain tasks. In this regard, for manufacturing plants in which heavy parts must be processed, disaster scenarios, service applications, etc. there is an ever-present need for interaction with the surrounding environment. Humanoid robots, physically similar to human beings, must fulfill a very important requirement: the robot must be able to move around its environment while maintaining its balance.

When a humanoid robot performs tasks and walks through plain, rough, or sloped terrains it has to be ensured that the robot will not fall over [1,2]. If there are obstacles placed in the robot environment and path re-planing is required [3,4], the normal step pattern must be changed while maintaining stability. Furthermore, prior to walking pattern generation, robot joint constraints, dynamic parameters (velocities, accelerations, etc.), and joint torques [5] have to be observed in real time so as not to overload the system and make the walking task viable.

In the case of the presence of human beings, unexpected disturbances can occur due to intentional or accidental interactions. In this situation, the robot is subject to an external force and the robot must counteract it to recover its balance status and prevent a fall [6,7]. A more complex situation comes when the robot is carrying an object or is collaborating with a human [8]. An unknown weight then has to be considered and the system model is completely different, taking the object as a part of its body. Each one of these situations leads to the use of one particular model of the robot, which takes into account different requirements from the surrounding environment, the mechanical distribution of the robot itself, etc.

The complex mechanical structure, high number of degrees of freedom, and control requirements of humanoid robots have led to the search for simplified models that enable the deployment of multiple tasks. However, the use of these models leads to the amplification of inherent inaccuracies of the humanoid robot system. The concept of the “simplified model” implies the assumption of errors to favor other aspects such as computing velocity, controllability, etc. The simplest model of a humanoid robot used in balance control is the inverted pendulum. It represents the location and movement of the center of mass (CoM) of the robot, which pivots around a support base thanks to a rotating joint. Due to its simplicity, it is easy to state that many inaccuracies are introduced and system features are omitted. For instance, the location at any time of the CoM depends on the posture of the robot and may coincide with the location represented by a pendulum model with a specific and fixed configuration.

**Figure 1:**
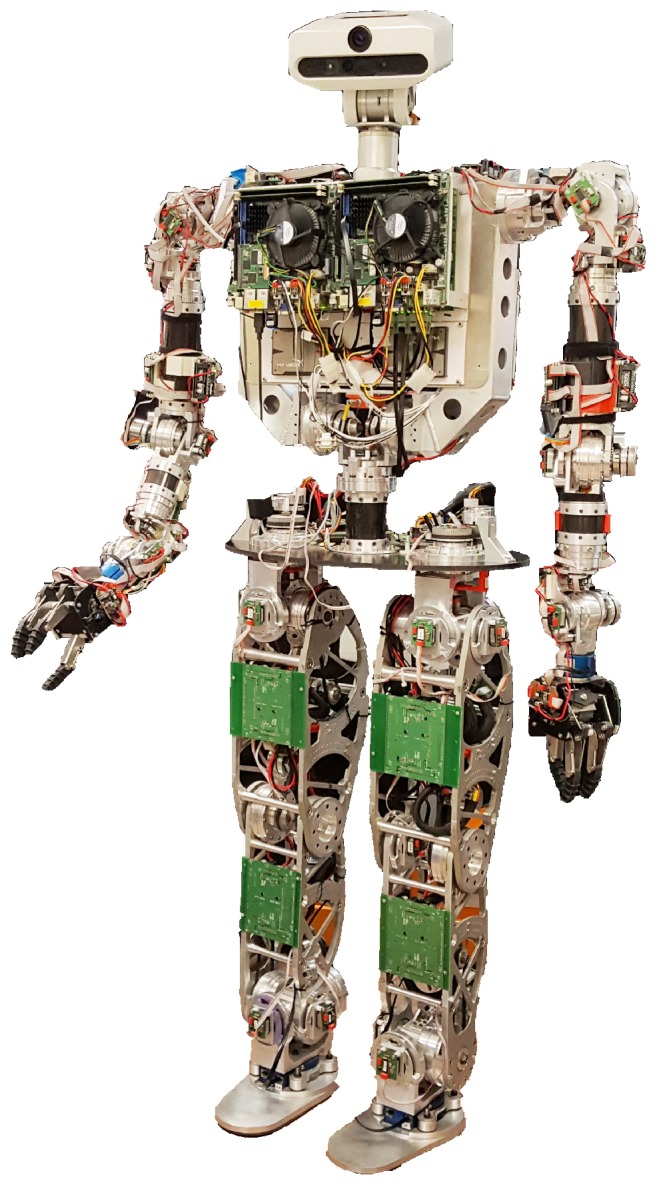
The Task Environment Operator (TEO) humanoid robot from the University Carlos III of Madrid.

Many improvements and new models have been developed in order to add more information about the robot body or to address the lack of information, such as in [9,10]. In addition, these new models can represent special behaviors or model special tasks [11,12]. This paper presents one of those improvements for dealing with robot inaccuracies such as material flexibility or component tolerances that are very difficult to model. System inaccuracies have been quantified and studied to experimentally model the robot response. Then, these errors have been modeled and, finally their effects reduced by introducing a counteracting action within a pendulum model, as will be described in following sections. By means of an error-scheduling method, the model parameters for control can be dynamically computed. This can be established as a methodology that can be applied to other robot control tasks. The experimental platform used in this work is the *Task Environment Operator* (TEO) humanoid robot from University Carlos III of Madrid [13], shown in Figure 1.

## 2. Background

To address complexity issues, the humanoid robot is usually represented by means of simplified models that enable an easy way of designing controllers. These models represent the kinematics and dynamics of the robotic system in action. Taking into account different parameters of the robot, such as the mass, the location of its CoM, inertia tensors, etc., many approximate models of the robot for each task context can be established. This work is focused in the study of simplified models applied in balance control and how inherent model errors can be overcome to improve robot operation. This background study is divided in the enumeration of some simplified models and how they are used in balance control.

### 2.1. Robot Simplified Models

**Figure 2:**
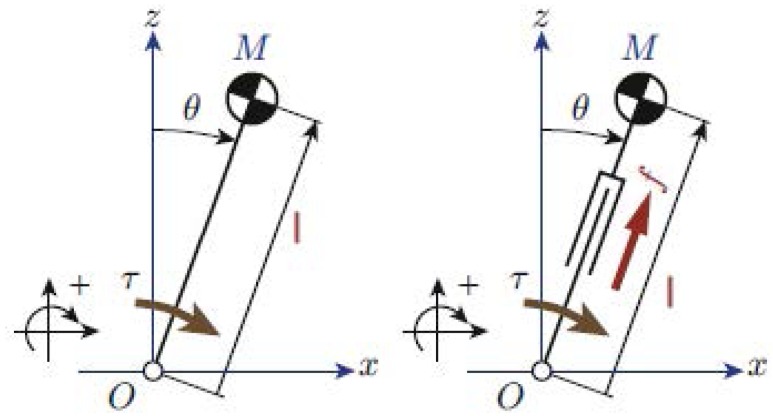
Basic inverted pendulum models in the x-z plane. One degree of freedom (**left**) and two degrees of freedom (**right**).

The simplest model for representing robot’s kinematics and dynamics is the two-dimensional inverted pendulum with one or two DoFs [14]. These models represent a concentrated CoM linked rigidly to the ground by one rotational joint like in Figure 2 (left), or including a linear joint like in Figure 2 (right).

**Figure 3:**
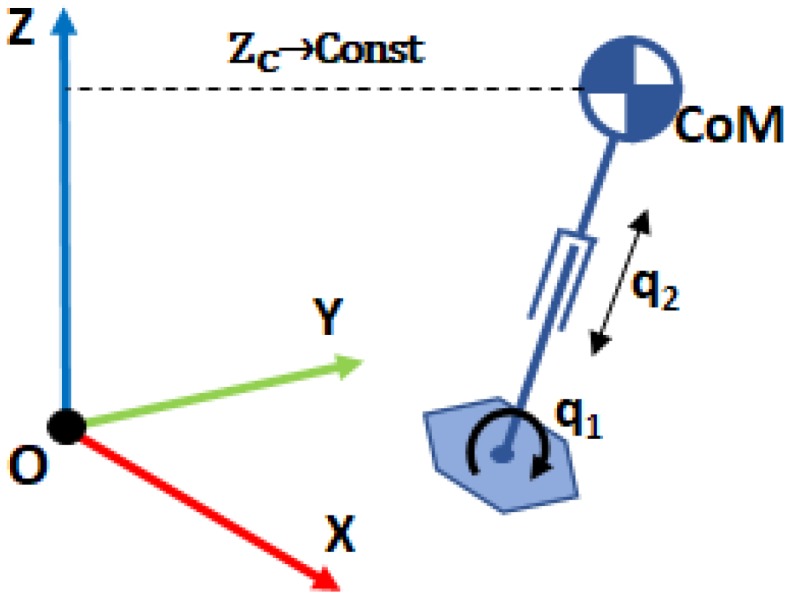
The Three-Dimensional Linear Inverted Pendulum Model (3DLIPM).

In the case of Figure 2 (left), the movement of the CoM is defined by the following equation:

(1) $$\tau =-ml^{2}\ddot{\theta }+mgl\sin\theta$$

where *m* is the mass of the CoM, *l* is the pendulum longitude, τ is the torque at the pivot point, and θ is the pendulum angle. However, this is a non-linear equation that makes its implementation in a robot controller more difficult. To overcome this problem it is assumed that θ is small enough to consider sinθ=θ. Then, the resulting model is one of the most famous models used in humanoid robotics. It is the Three-Dimensional Linear Inverted Pendulum model (3DLIPM) shown in Figure 3, which was proposed by Kajita [15].

Then, Equation (1) becomes (for the 2D case in plane x-z):

(2) $$\tau =-ml^{2}\ddot{\theta }+mgl\theta$$

with the z-coordinate movement constrained to an horizontal plane (Figure 3):

(3) $$z=z_{c}$$

**Figure 4:**
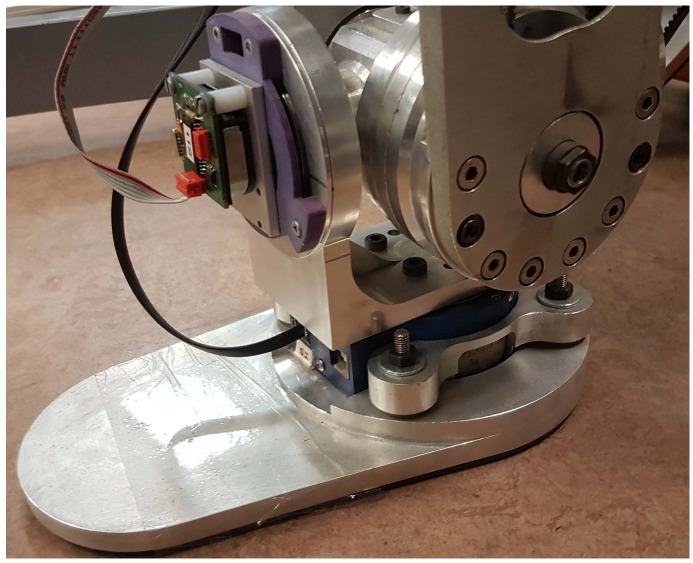
The TEO robot’s ankle joints with the force–torque sensor.

The main advantage of the 3DLIPM is that the linear equations are very easy to program in a computer. It is mainly used for walking pattern generation and balance control. The application of this model for balance control is possible when the ground reaction (vertical force) and torques in the robot’s ankle joint (which correspond with the point *O* of the model) can be measured. This has been achieved by the use of force–torque (F-T) sensors at the ankle level, such as JR3^TM^ sensors assembled in TEO robot feet (Figure 4).

**Figure 5:**
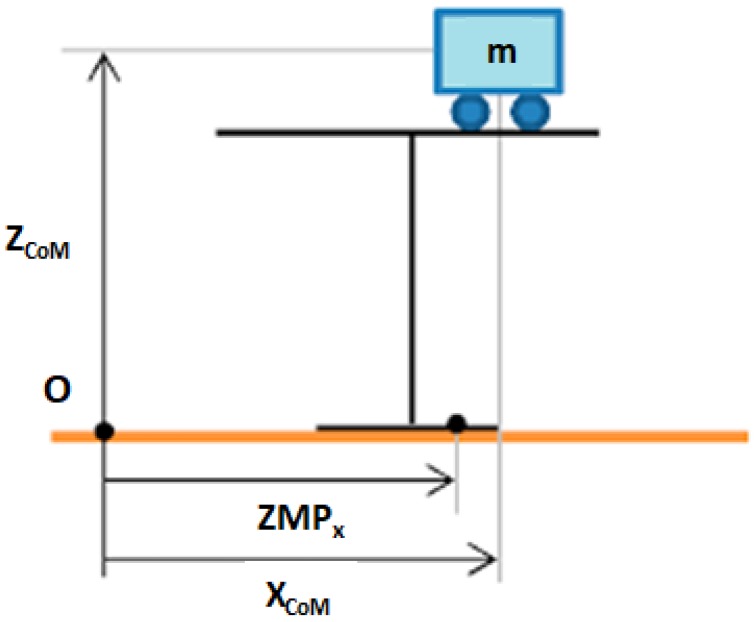
The cart-table simplified model. ZMP: zero moment point.

However, the 3DLIPM does not provide information about body accelerations and inertias, which is very useful for biped robots during dynamic walking tasks. This issue was resolved with the development of the cart-table simplified model (Figure 5). In this case, the information needed by the model is provided by inertial measurement units (IMUs) which sense velocities and accelerations of the robot body.

The cart-table model and 3DLIPM are the most used simplified models in balance control. Nevertheless, other researchers employ multi-link models, where they use precise knowledge about the dynamics of each robot link [11,16].

### 2.2. Zero Moment Point and Balance

The study of humanoid robots balance has been supported by the simplified models described earlier. Many tools have been developed to describe the kinematic and dynamic behavior of a humanoid when it performs tasks. Taking in account that one of the main goals of a humanoid robot is to achieve stable walking behaviors, these tools have been widely studied in this field.

**Figure 6:**
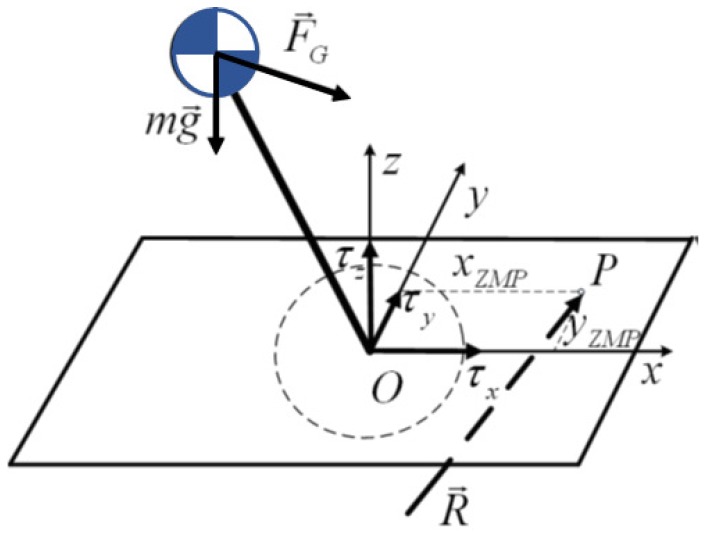
The ZMP location (*P*).

The development of humanoid balance control architecture is mainly related to the study of two specific reference points. The first one is the center of mass used to model the humanoid body, as described in the previous subsection. However, the CoM does not provide useful information about the body balance status. The zero moment point (ZMP) introduced by Vukobratovic in [17] is the first and the main tool developed for describing the body’s equilibrium. The ZMP was defined firstly as the point in the support base, usually the ground, where the resulting torque caused by all forces exerted over the robot’s body is equal to zero. Figure 6 illustrates the ZMP location *P*, and Equation (4) defines it mathematically.

(4) $$P_{x}=-\frac{\sum x\cdot F_{z}}{\sum F_{z}}$$

In Equation (4), for the coordinate *x*, the sum of the torques produced by the mass of each link of the body due to gravity is divided by the sum of reaction forces. For a static posture, if the value of the ZMP coordinate lies inside the support polygon of the robot, the balance of the robot can be guaranteed. However, when the ZMP is at the edge of the support area, the humanoid body can lose balance and fall down.

**Figure 7:**
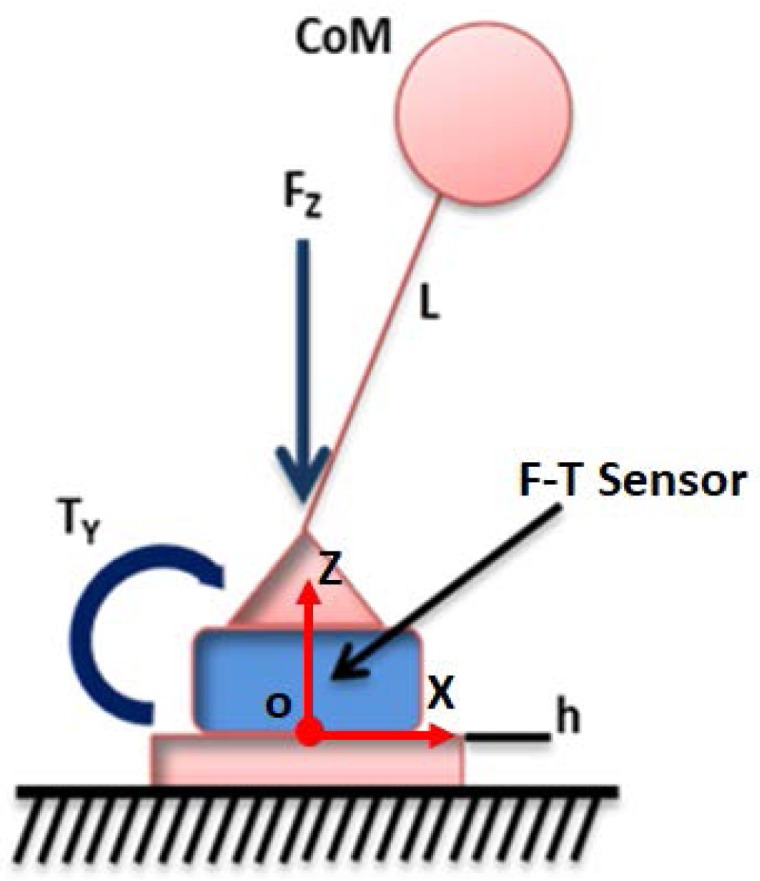
The LIPM model with an F-T sensor between the sole and ankle joint.

The computation of the ZMP depends on the posture of the robot and the location of the CoM of each limb. Due to this, ZMP calculations have an advantage in representing the robot body as a simplified model for two main reasons. The first one is the simplicity of the equations used for ZMP computation. The second reason is the possibility of using F-T sensors to measure all the forces and torques needed for ZMP computation. The model applied in this work is the 3DLIPM modified to match with the TEO robot structure, as can be observed in Figure 7.

When a biped robot is supporting its body on one foot, the robot ankle is considered the pivot point connected to the robot’s CoM by means of a massless leg. The simplest model only considers the gravitational force exerted on the mechanism, and the pendulum motion is represented by Equation (2). According to [18], the ZMP equation in the sagittal plane obtained from the LIPM when the robot is standing on one foot is as follows:

(5) $$x_{ZMP}=-\frac{\tau_{y}+hF_{x}}{F_{z}}$$

where $\tau_{y}$ is the torque at the pivot point around the *y* axis, $F_{x}$ is the measured force in the *x* direction, and *h* is the distance from the ground to the location of the sensor (generally the sole height). However, when the robot stands with double support (with both feet flat on the ground), the ZMP obtained from each foot is used to compute the global ZMP [14]:

(6) $$x_{ZMP_{DS}}=-\frac{x_{ZMP}^{R}\cdot F_{z}^{R}+x_{ZMP}^{L}\cdot F_{z}^{L}}{F_{z}^{R}+F_{z}^{L}}$$

where the upper index *R* represents the right foot and *L* represents the left one. When the robot is in the double-support phase and two pivot points at the ankle joints exist, the inverted pendulum can be used. If the movement is in the sagittal plane, the robot behaves as a single inverted pendulum because both ankles have the same movement along the *x* axis.

### 2.3. Balance Control

One of the main skills defining the human being is the capacity to walk upright. This is one of the main features that a humanoid robot must achieve. The key issue is the balance of the upright posture to avoid falls during a walking task or while standing still. The use of the simplified models of the body and tools such as ZMP enables the deployment of stabilizers to maintain equilibrium.

**Figure 8:**
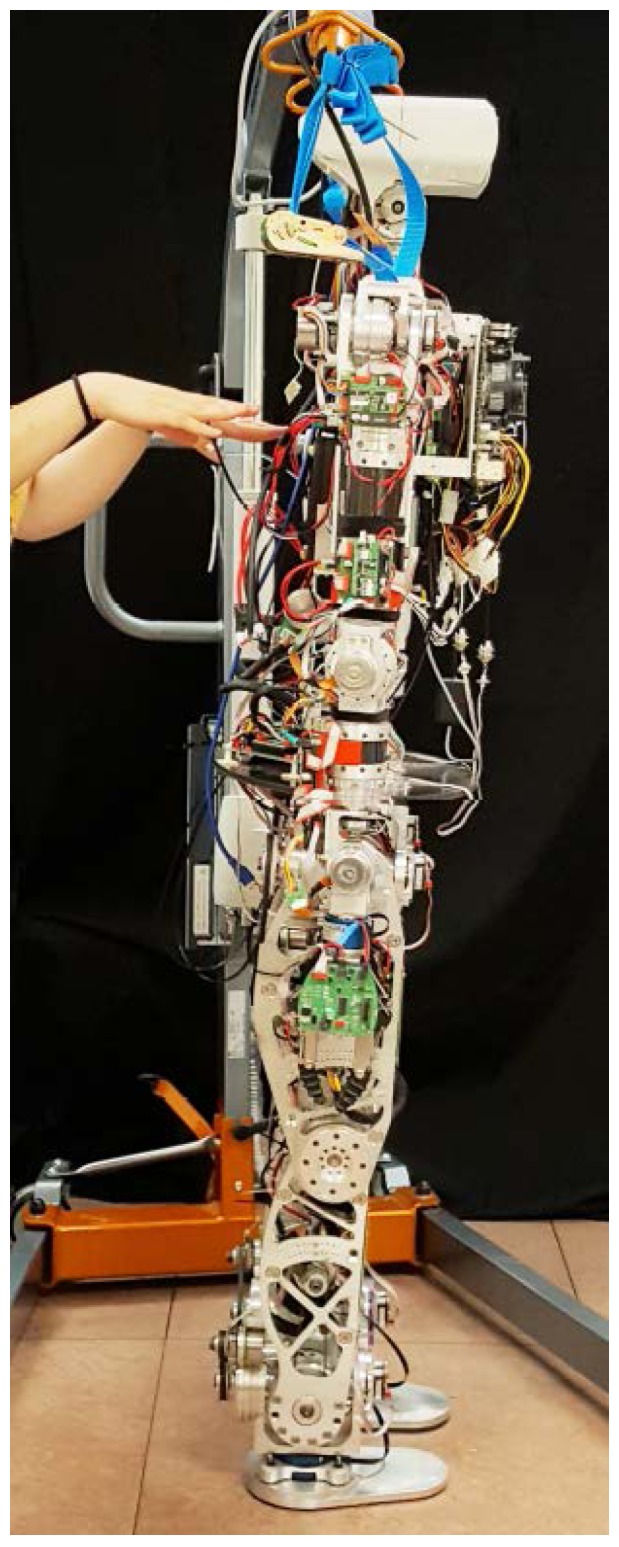
Experiment on applying force to the TEO robot.

Before performing a walking task, the humanoid robot must maintain a stable upright posture. In this situation, unexpected disturbances are the first issue the robot must deal with to develop a balance control architecture. Hence, achieving this upright stable posture is the first stage in developing an stabilizer. One of the main techniques for the development of a balance control architecture is based on push-recovery experiments, as shown in Figure 8. The robot must deal with unexpected disturbances represented through the forces applied to it. If unexpected disturbances appear, and depending on the intensity level of the disturbance, different control strategies can be set [19]. These include ankle, hip, and step strategies. For low-intensity disturbances, the body can be considered as a nearly single stiff pendulum, where balance adjustments are mainly made in the ankle joints of the robot [12]. The hip strategy is applied when the external disturbance increases and the ankle strategy is not sufficient for keeping balance. When using this strategy, the robot can move its hip independently or in combination with the ankle strategy. Then, the robot model has to be modified, considering a double inverted pendulum [19,20]. The double inverted pendulum consists of a upper link and a lower link, where each single pendulum has an influence on the other one. The step strategy is only used when postural corrections become insufficient and the base of support must be adjusted. Taking this in account, the very first step in developing an stabilizer is to deploy the control system for each strategy, starting from the ankle one.

**Figure 9:**
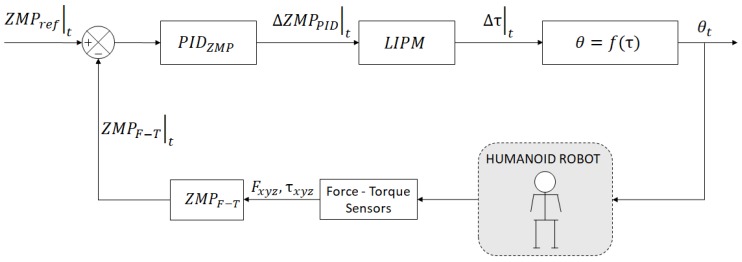
Basic ZMP position controller using the LIPM model.

Balance control with the ankle strategy concept is applicable both to standing in upright posture and to walking tasks. In both situations, the robot is modeled as an inverted pendulum. The disturbance is a force applied to the CoM of the model. This force can lead the ZMP to be out of the support polygon and the robot will lose balance. Then, the robot must counteract this disturbance by applying a torque in the ankle joints, trying to maintain ZMP inside the support area. This kind of control is called *ZMP control by ankle torque* [21,22]. Finally, the ankle’s torque is transformed into angle commands θ=f(τ) and they are sent to the robot. This is represented by the control architecture depicted in Figure 9.

However, traditional PID control relies on the proper the proper selection of values to be used for the proportional (P), integral (I), and derivative (D) constants for a linearized working point [23]. If the process is non-linear, the control designer must then continuously evaluate it and tune the constants. Instead of using PID controllers, model-based controllers are able to learn how a process responds to changes, and in turn, they can automatically make the tuning adjustments that would traditionally be manual.

## 3. Problem Statement

However, there are many errors that the balance control system must deal with. Simplified model control approaches always introduce errors. The pendulum mathematical model is not linear, but ZMP equations are obtained from a linear pendulum. When the angle of the pendulum is small enough, it is assumed that sinθ=θ, which introduces an error to the system. The mass of the center of gravity (CoG) is also an approximated value of the whole robot mass, and even its location can change. When all these assumptions are added together, errors in the system become remarkable.

Also, there can be measurement deviations in the F-T sensors due to calibration errors, or in analogue-to-digital data conversions. Other systematic errors, such as the flexibility of the structure (due to the height of the robot), looseness between mechanical parts (as transmissions or unions of pieces), and small irregularities in the ground, are usually not considered. All of these errors lead to an increase in the control effort and make the control tuning task more difficult.

**Figure 10:**
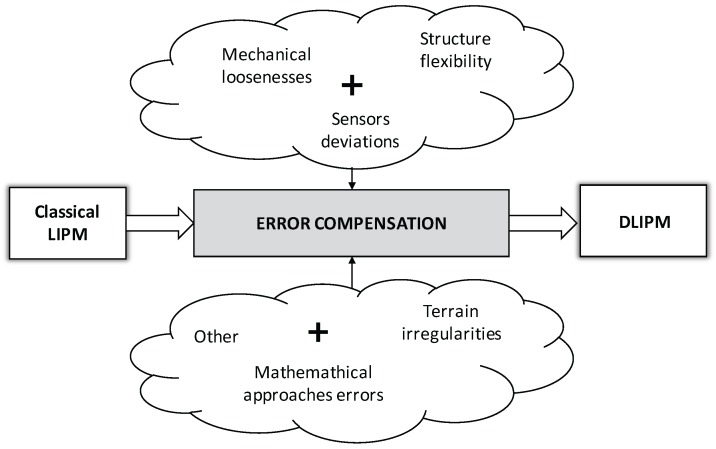
Error compensation diagram. From classical LIPM to new DLIPM.

The aim of this work is to improve the ZMP control system described in Figure 9, and the Dynamic LIPM (DLIPM) is proposed. This model will include the errors depicted in Figure 10 and more. The procedure to model this error is based on push-recovery experiments in which the ZMP is computed thanks to the measures provided by F-T sensors. Then, the real ZMP is compared with the planned ZMP, obtaining the error. Finally, the error is introduced in the model as a fictitious force that modifies the inverted pendulum model behavior.

From the control point of view, real humanoid mechanisms are slightly flexible [21]. Usually the flexibility is closely related to the robot height and, although robot designers try make stiff structures, it is impossible to eliminate the flexibility altogether. Because of this, the humanoid robot exhibits the characteristics of a lightly damped structure. For example, in a static case when the ankle joint is under position control, a pushing external force can easily excite an oscillation. This oscillation exists even when the position error in every joint is zero. In addition, there are other error sources that have an influence on the correlation of the robot with the model (Figure 10). However, it is very difficult to identify and define these errors mathematically.

**Figure 11:**
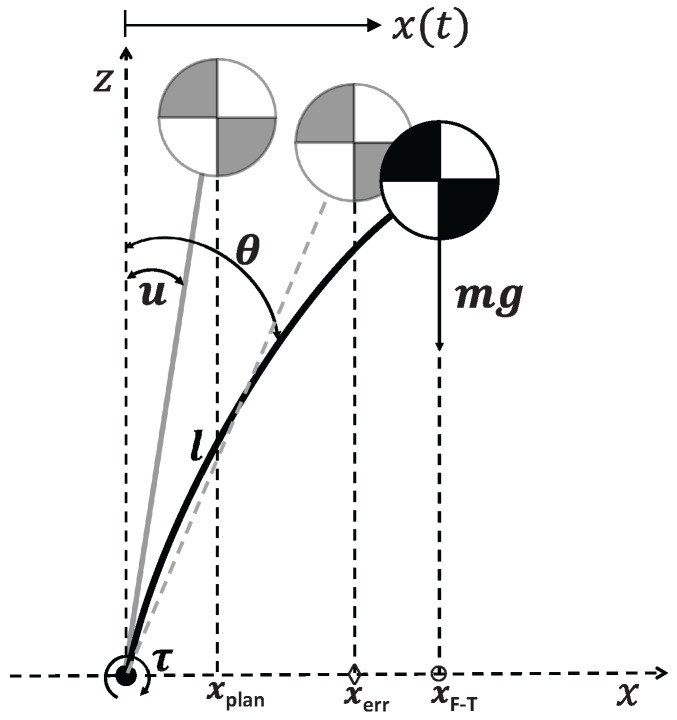
Single inverted pendulum model including the robot’s flexibility [21].

The existence of these errors has a great influence on the ZMP computation and, as such, on the balance control system. Figure 11 illustrates how robotic system inaccuracies and other error sources affect to the location of the ZMP. In this example, *u* denotes the model angle expected caused by the commanded joint torque. The expected ZMP would be represented by $x_{exp}$. If we consider only the error introduced by the robot flexibility, the ZMP location would be the one represented by $x_{err}$. Nevertheless, the real ZMP computed using the forces and torques measured is $x_{F-T}$.

Then, the problem is the mismatch between the expected or planned ZMP and the real ZMP measured with the F-T sensors. In order to reduce this gap, this work proposes a model improvement to more closely represent real robot behavior. Furthermore, a ZMP control architecture for keeping balance can improved as well.

## 4. Methods and Experimental Procedure

The error has been modeled using the information of the F-T sensors installed in the ankles of the robot. All the effects caused by any disturbances are reflected in the forces and torques measured by the sensors. In this way, it is necessary to separate the information related to the inaccuracies and the information related to the expected behavior. Two assumptions need to be made before performing this procedure. The first one is the need to establish the inverted pendulum model parameters: CoM location and mass. They come from the robot design but they are not completely accurate because of the differences between CAD designs and the real implementation. The correction of this parameters using the real robot is not possible, so the use of the theoretical values is assumed. The second assumption is related to the planning of the balance control task. Taking into account the established model, the ZMP location can be planned. That is, ZMP location can be pre-planned in order to always remain inside the support polygon. The balance plan should resemble the reality in order to reduce the effort of the control system. This means that lower gains will be needed to adjust the control system.

The method used to develop the new improved model is as follows: based on open-loop system push-recovery set of experiments, the measurements of the F-T sensors are captured and processed. Then, with this information, the real ZMP $x_{F-T}$ is computed and compared with the expected ZMP $x_{exp}$. The difference between them is modeled and one equation describing this error is obtained. The modeled error is included in the original model as a fictitious force that corrects the difference found. Once the new model has been obtained, the new planned ZMP behavior is close to that of the measured ZMP.

### 4.1. Study of the System Response

**Figure 12:**
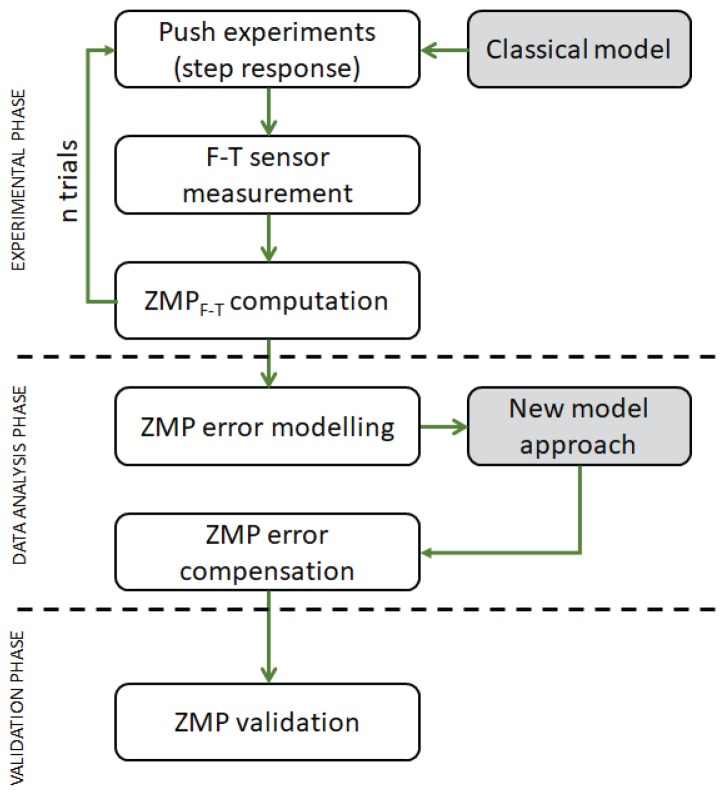
Experimental procedure diagram.

To introduce all the errors mentioned before into the TEO simplified model, the procedure summarized in Figure 12 has been followed. This procedure has been divided in three phases: the experimental phase, data analysis, and validation of results. This procedure has allowed to obtain a set of data from the F-T sensors to be used in the generation of the new custom model.

The first stage of the procedure is to fix the inverted pendulum model parameters with the characteristics of the TEO humanoid robot. The weight of the is 62.6 kg and the height from the ground to the CoM is 0.893 m (the pendulum length). Then, the movement of the robot caused by a pushing force was tested. The effect of this force is a variation of the ZMP location depending on its intensity. It is important to remark that the robot does not keep the initial value of the ZMP during each experiment. That is, it does not recover the initial posture. This behavior is similar to the study of the response of a system with an step angular input in the ankle joint. After a set of trials, the ZMP error has been modeled as the difference between the expected and the measured value. The dynamic behavior of the ZMP variation has also been studied. The last phase of this procedure consisted of the validation of the new model within the new control architecture.

**Figure 13:**
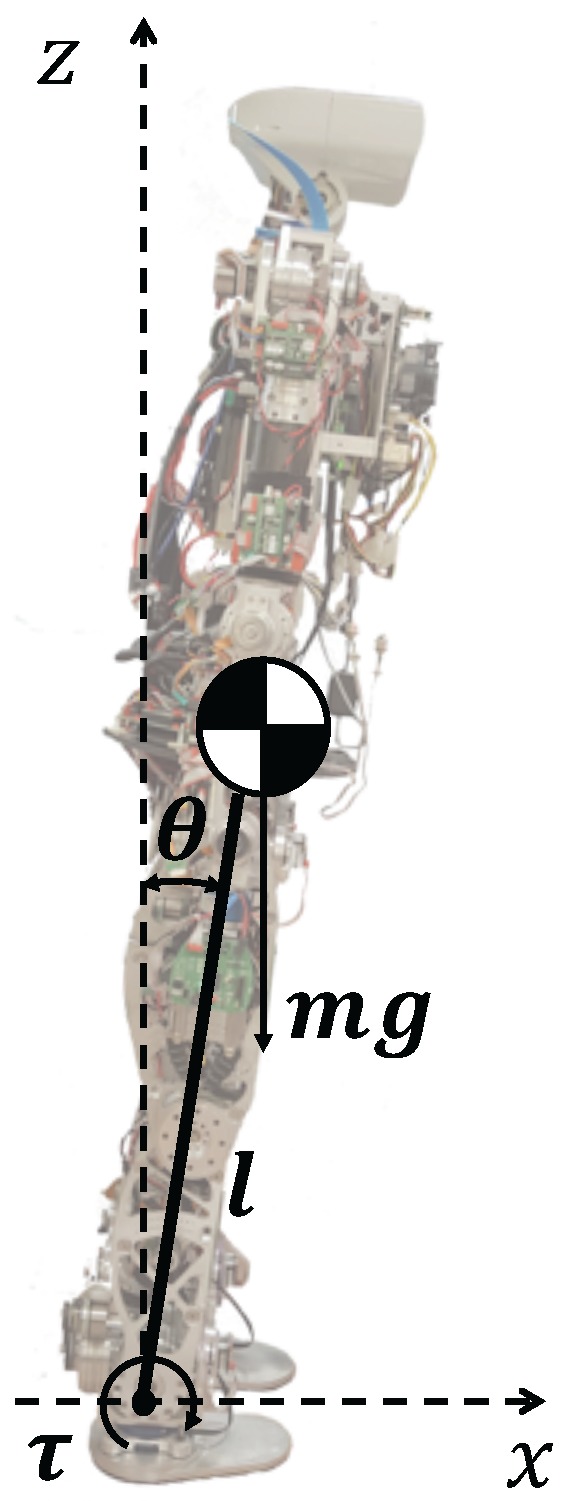
Experimental setup of the TEO robot.

To illustrate the method, only the results from the sagittal plane (x-z) of the robot are presented because the experimental methodology for the frontal plane (y-z) is the same and similar results were obtained. This study and methodology are applicable to any other spatial direction. In this way, the experimental setup is represented by Figure 13. The robot is in a flat ground environment with both feet on the ground (double support). Therefore, the support area includes the robot footprints and the common tangents between them.

**Figure 14:**
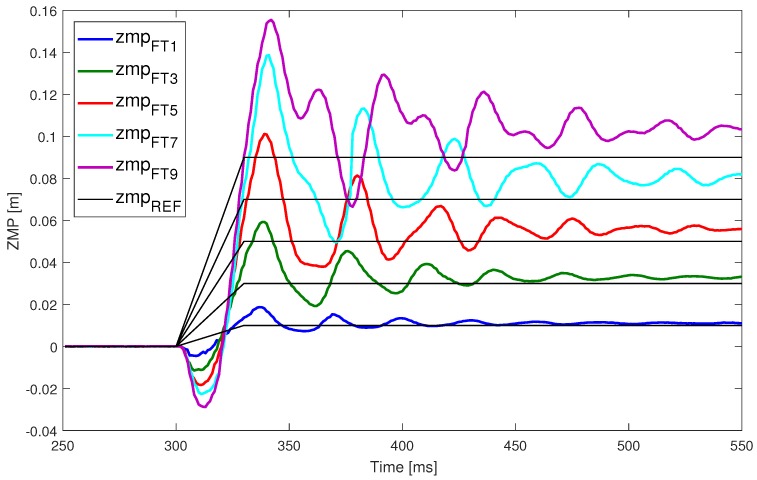
Step response experiments.

The results of a set of trials are shown in Figure 14. This figure represents the measured ZMP (the oscillating signals) and the expected ZMP (the step form signals). Each pair of ZMP signals (oscillating step) corresponds to a specific pushing force applied to the robot. If we examine each pair, some conclusions can be extracted. Larger disturbances imply a further location of the ZMP from its origin, making the robot more unstable because the ZMP is closer to the support polygon edge. This means that the angle from the model that is commanded to the robot is larger, and the errors have more influence— mainly with respect to robot flexibility and mechanical tolerances. For this reason, the steady state error is greater as well. Furthermore, the system has a higher initial oscillating response, which is not desirable when the ZMP is located near the edge of the support polygon.

This dataset is the basis for developing an improved ZMP control without the necessity for low-level position or torque controller parameter tuning. The objective of next steps is to obtain a transfer function modeling the behavior of the ZMP. The resulting transfer function, that models ZMP deviations, will be added to the classic LIPM with two main purposes: the elimination of the steady state error and the reduction of transient oscillation and overshooting.

### 4.2. Dynamic Linear Inverted Pendulum Model

**Figure 15:**
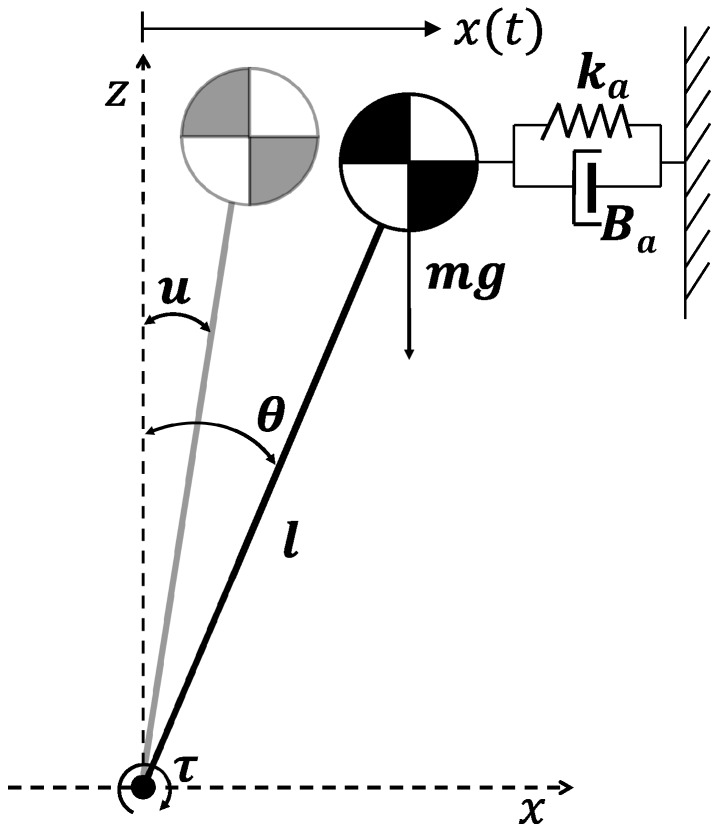
Proposed compensated inverted pendulum model.

To accomplish the ZMP control requirements (settling time and overshoot level), this work proposes an improvement model derived from the classic LIPM. The objective is to modify the initial model, adding a system that represents the errors of the real robot obtained from experimentation. Then, the measured balance parameters will have less of a deviation from planned ones, and the control response can be reduced. Figure 15 represents the complete model in which a spring $k_{a}$ and a damper $B_{a}$ have been added to the initial inverted pendulum model. These mechanical models try to compensate the steady state response ($k_{a}$) and the transient response to limit oscillations ($B_{a}$).

Then, the equation of motion of the model shown in Figure 15 is given by:

(7) $$\tau =-ml\ddot{x}\left(t\right)-B_{a}l\dot{x}\left(t\right)-k_{a}lx\left(t\right)+mgx\left(t\right)$$

where x(t) is the CoM movement, *m* is the pendulum mass located at the CoM, *l* is its longitude, $k_{a}$ is the spring constant, and $B_{a}$ is the damper constant. The displacement of the CoM is small enough to assume sinθ=θ. Then, Equation (7) becomes:

(8) $$\tau =-ml^{2}\ddot{\theta }\left(t\right)-B_{a}l\dot{\theta }\left(t\right)-k_{a}l\theta \left(t\right)+mgl\theta \left(t\right)$$

Torque can be also obtained from the ZMP measurement as:

(9) $$\tau =-x_{FT}\cdot mg$$

where $x_{FT}$ is the measured ZMP from the sensors. Combining both equations we obtain:

(10) $$-ml^{2}\ddot{\theta }\left(t\right)-B_{a}l\dot{\theta }\left(t\right)-k_{a}l\theta \left(t\right)+mgl\theta \left(t\right)=-x_{FT}\left(t\right)mg$$

Finally, the transfer function obtained from Equation (10) is:

(11) $$\frac{\Theta (S)}{X(S)}=\frac{\gamma }{S^{2}+\alpha S+\beta }$$

where $\gamma =g/l^{2}$, $\alpha =B_{a}/ml$, and $\beta =(k_{a}-g)/l$. In the steady state, when time goes to infinity, the DC gain of the system is represented by Equation (12), that only depends on the $k_{a}$ parameter. This one is in charge of eliminate the static error.

(12) $$K_{DC}=\frac{\gamma }{\beta }$$

### 4.3. Steady State ZMP Error Characterization

**Figure 16:**
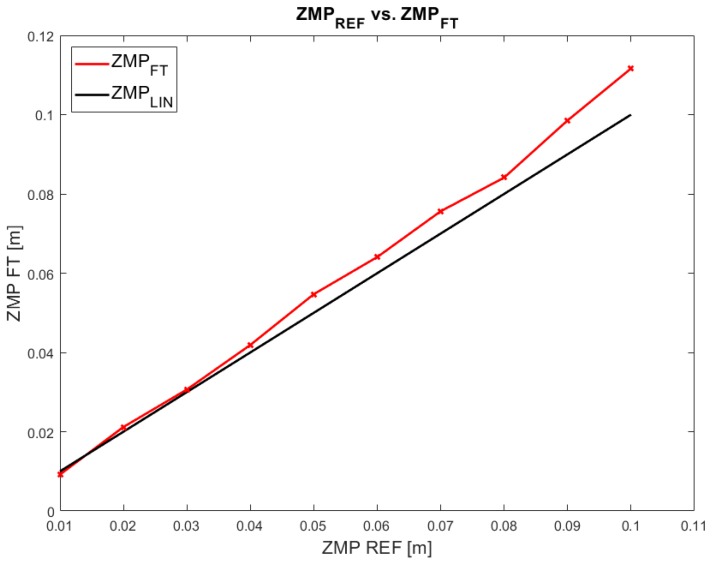
Comparison of experimental $ZMP_{ref}-ZMP_{F-T}$ steady state values.

The next step is to characterize the deviation of the ZMP. Even though the ankle position control succeeds, the ZMP measurement presents deviations. This means that the angular controller of the ankle joint works properly but the measured ZMP value has a deviation from the planned or expected value. From the trial dataset depicted in Figure 14, the deviation of the ZMP can be determined. Figure 16 represents this deviation of the $ZMP_{F-T}$ (output) from the $ZMP_{ref}$ (input). The relation between the expected ZMP value and the measured one should be linear but the figure demonstrates that the real values do not match with the expectations.

The deviation in each test point is used for fitting to the second-order polynomial Equation (13). This equation represents the real ZMP $x_{F-T}$ measured by the ankle sensors:

(13) $$x_{F-T}=a\cdot x_{ref}^{2}+b\cdot x_{ref}+c$$

where a=0.834, b=1.024, and c=−0.0004.

This equation represents the steady state error of the open-loop system for each working point. Equations (12) and (13) are the basis for planing the evolution of the joint angle and, therefore, ZMP location. Once the static error has been minimized, the transient response must be optimized to reduce the level of oscillations.

### 4.4. ZMP Transient Response Characterization

**Figure 17:**
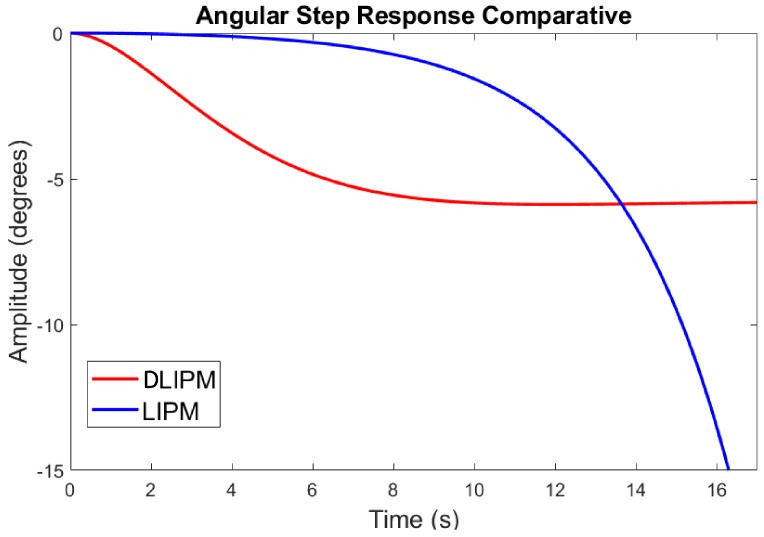
Angular step response for the LIPM and DLIPM.

The linear inverted pendulum is inherently unstable. It is necessary to develop a controller to stabilize it against any kind of disturbance. Meanwhile, the angular response of the inverted pendulum is infinite, and higher order systems can present stable behaviors. Figure 17 shows the comparison of the LIPM vs. DLIPM transfer functions in response to a simulated step perturbation. This behavior also means that the dynamic parameters can be adjusted to higher values in the DLIPM case than in a controller including the LIPM model, with a greater margin of adjustment.

**Figure 18:**
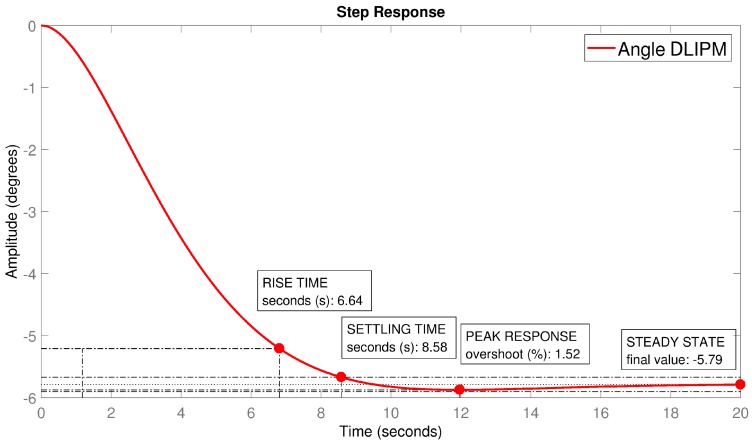
Angular step response of the DLIPM.

The behavior of the humanoid robot system acting like an inverted pendulum has been demonstrated as an under-damped system. By selecting appropriate gain and dynamic parameters it is possible manipulate the overall response of the system, reducing the overshooting and oscillations of the system. The ZMP oscillations have higher values when the location of the ZMP is further from the origin and, in addition, when the input angles have a high variation. This relation between ZMP and ankle angle allows the for a reduction of the oscillation level by means of angle planning. In Equation (11), dynamic parameters can be configured, for example, to limit the overshooting level. These dynamic parameters can be obtained from α that is related to $B_{a}$. Figure 18 shows the signal obtained from the simulation of a disturbance causing a ZMP variation of 9 cm. The dynamic parameters were designed to obtain an over-damped response (ξ=0.8, $\omega_{n}=0.4376$).

Then, selecting the proper parameters it is possible to modulate the dynamics of the robot and reduce undesired oscillation levels on the robot.

### 4.5. ZMP Control

Classical control architectures, such as the one shown in Figure 9, are based linearized controllers around a working point. This means that the controller has almost no error at this working point, and it commit more error as the control target moves further than this point. In this work, a non-linear solution is proposed based on *gain scheduled matching*. The main goal is to dynamically select the most appropriate parameters for multiple working points of the controller. That is, the dynamics of the DLIPM model depend on the actual behavior of the robot and not on pre-computed static parameters. In control theory, a gain-scheduled controller is a system control architecture in which its gains are automatically adjusted as a function of time, operating condition, or plant parameters [16]. Gain scheduling is a common strategy for controlling systems in which its dynamics change with such variables. Typically, gain-scheduled controllers are fixed single-loop or multi-loop control structures that use lookup tables to specify gain values as a function of the scheduling variables. For tuning purposes, it is convenient to replace lookup tables with parametric gain surfaces, such as fuzzy surfaces [24,25]. A parametric gain surface is a basis function expansion in which its coefficients are tunable. For applications where gains vary smoothly with the scheduling variables, this approach allows for the tuning of a few coefficients rather than many individual lookup-table entries, drastically reducing the number of parameters. This approach also provides explicit formulas for the gains, and ensures smooth transitions between operating points.

**Figure 19:**
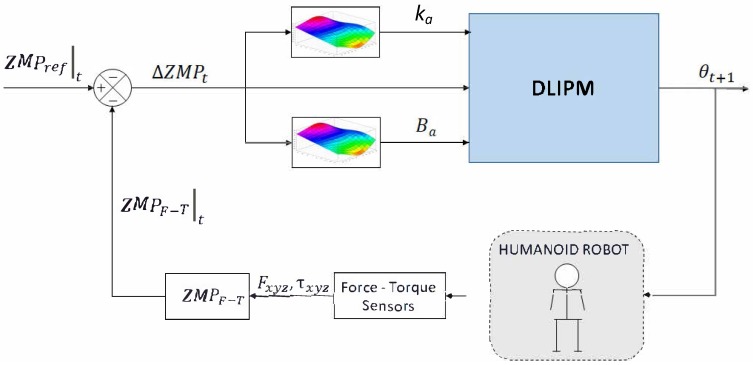
The TEO ZMP controller.

The control architecture is presented in Figure 19, similar to the human-inspired control architecture presented in [26]. In this case, there is a preprocessing module for control parameter planning. Depending on the input *u*, the appropriate values for $k_{a}$ and $B_{a}$ can be selected. Then, these parameters are used for computing the values of the coefficients of the DLIPM’s transfer function. These parameters customize and modify, for each control state, the dynamics of the DLIPM model. Finally, the DLIPM module outputs the ankle angle to be commanded to the robot. The angular transition is in this case smoother than that obtained with other approaches.

### 4.6. Experimental Validation

To check the feasibility of the proposed system, it was tested experimentally by performing a set of trials for capturing the response of the control system against a variation on the ZMP target. The DLIPM state space model was customized with the parameters for each ZMP target, following the step pattern. Then, the outputs of the model were the customized angle commands following the ZMP planning. Table 1 shows numeric results of the ZMP locations, comparing the values obtained from the classical approach and the DLIPM approach. It can be observed that the static error is reduced in each working point. Even in the most critical ZMP location (ZMP=10 cm), the error was reduced by more than 80% when comparing the obtained ZMP measurements using the classical LIPM and the compensated model proposed in this paper.

**Figure 20:**
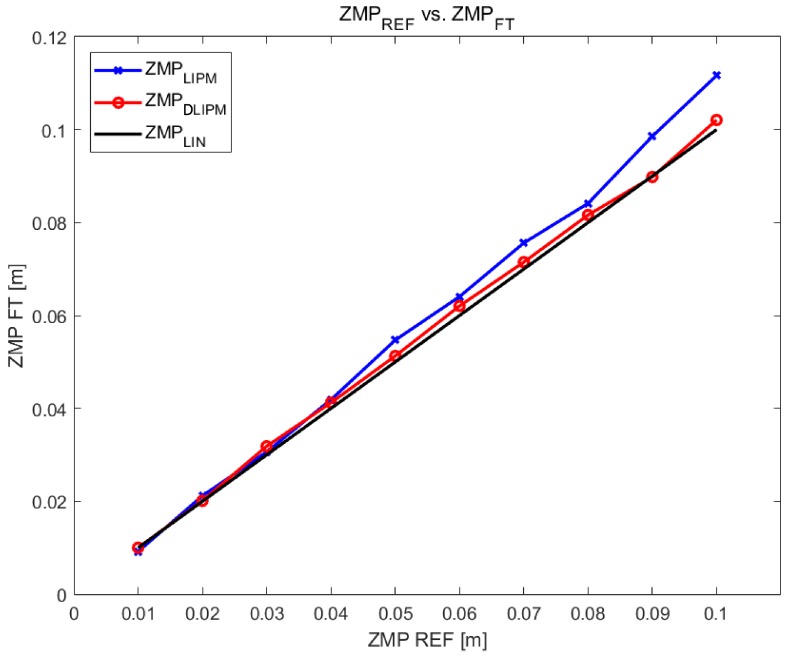
ZMP comparison using the LIPM and DLIMP.

Data from Table 1 have been depicted in Figure 20. It can be observed that the error in the classical system is higher when the ZMP location is further from the initial zero position ($ZMP_{LIPM}$). Furthermore, the DLIPM curve ($ZMP_{DLIPM}$) is better adjusted to the desired linear response ($ZMP_{LIN}$).

**Figure 21:**
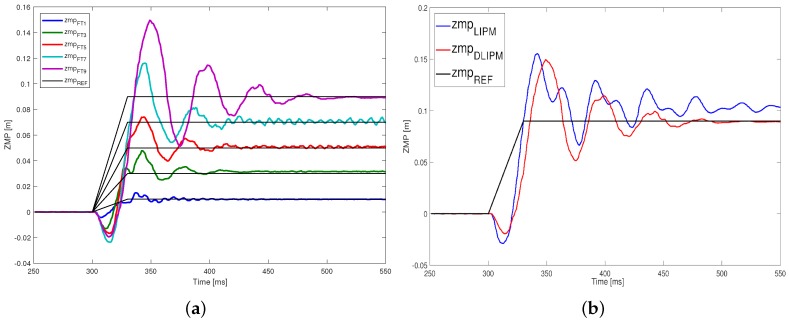
Comparison of ZMP step responses. (**a**) Step response experiments on DLIPM; (**b**) Comparison between the LIPM and DLIPM for ZMP = 9 cm.

With respect to the dynamic response of the system, Figure 21 depicts the results from all the trials performed. Comparing this figure with Figure 14, it is easy to observe that the level and the duration of oscillations have been reduced. While the overshooting has similar levels in some experiments, the state of the robot is stabilized in general earlier than the classical architecture.

## 5. Conclusions and Future Works

Humanoid balance control is based on the knowledge of certain equilibrium indicators. These parameters are materialized in mathematical models that represent simplifications of humanoid body behavior. The less simplified the model is, the more accurate the control performance; however then the computational complexity is greater. Classical simplified models, such as the LIPM, have a high level of simplification. The walking behavior and balance can be modeled, however approximation errors are introduced. On the other hand, the robot mechanics and electronics have inherent inaccuracies that are added to those of the model. This work has presented one method to modify the humanoid robot model to reduce these inaccuracies and to improve the balance control system. The experimental procedure, founded in push-recovery trials, has been used to determine the steady state error and the dynamic response of the system. This procedure can be applied to any kind of humanoid robot because it is independent of the system and it is able to characterize any kind of inaccuracy.

The resulting model, named here as DLIPM, is the basis for implementing a model-based balance controller. Linear balance controllers based on the use of these simplified models need very precise and complex tuning to find the optimal control parameters. Furthermore, these kind of controllers are designed to operate around a working point with minimum error. Nevertheless, the balance architecture proposed using the DLIPM has been conceived to operate in multiple working points, minimizing the error in each one. The DLIPM is a template that must be fulfilled with the proper parameters for each specific working point, which are related to the balance status of the robot (ZMP). These parameters define two aspects: the evolution of the ZMP between two consecutive postures, and the level of error in each ZMP. In the first case, a smoother trajectory between postures has been achieved, with the number of undesired oscillations reduced, especially in critical ZMP locations. In the second case, the error between the desired ZMP location and the measured ZMP has been reduced. These results are shown in Table 1.

Currently, the described work deals with humanoid robot modeling in a laboratory environment with flat surfaces. The next step is to extend the procedure to models applied to other robot behaviors, such as walking on uneven surfaces. Moreover, new improvements are needed to evaluate the influence of the upper body movement or the behavior of the control system when carrying objects.

## Figures and Tables

**Table 1:** ZMP comparison using the LIPM and DLIPM.

${\mathrm{ZMP}}_{\mathrm{REF}}$ (m)	${\mathrm{ZMP}}_{F-T}$ (m)			
	LIPM Model	% Error	DLIPM Model	% Error
0.00	$5\times 10^{-5}$	0.0	$2\times 10^{-7}$	0.0
0.01	0.0092	8.2	0.0100	0.0
0.02	0.0211	6.0	0.0201	0.5
0.03	0.0306	2.2	0.0310	3.3
0.04	0.0419	4.8	0.0412	3.0
0.05	0.0547	9.4	0.0512	2.4
0.06	0.0641	6.8	0.0620	3.3
0.07	0.0756	8.0	0.0714	2.0
0.08	0.0841	5.2	0.0816	2.0
0.09	0.0902	9.5	0.0989	0.2
0.10	0.1116	11.6	0.1020	2.0
